# The Long-Term Effects of Neonatal Inflammatory Pain on Cognitive Function and Stress Hormones Depend on the Heterogeneity of the Adolescent Period of Development in Male and Female Rats

**DOI:** 10.3389/fnbeh.2021.691578

**Published:** 2021-07-21

**Authors:** Irina P. Butkevich, Viktor A. Mikhailenko, Elena A. Vershinina, Gordon A. Barr

**Affiliations:** ^1^Laboratory of Ontogenesis of the Nervous System, Pavlov Institute of Physiology, Russian Academy of Sciences, Saint Petersburg, Russia; ^2^Department of Information Technologies and Mathematical Modeling, Pavlov Institute of Physiology, Russian Academy of Sciences, Saint Petersburg, Russia; ^3^Department of Anesthesiology and Critical Care Medicine, The Children’s Hospital of Philadelphia and the Perelman School of Medicine, Philadelphia, PA, United States; ^4^Department of Psychology, University of Pennsylvania, Philadelphia, PA, United States

**Keywords:** neonatal pain, corticosterone, adolescence, spatial memory, sex differences, spatial learning

## Abstract

Exposure to stress at an early age programs the HPA axis which can lead to cognitive deficits in adults. However, it is not known whether these deficits emerge in adulthood or are expressed earlier in life. The aims of the study were to investigate (1) the immediate effects of early injury-induced stress in one-day-old (P1) and repeated stress on at P1 and P2 rat pups on plasma corticosterone levels; and (2) examine the subsequent long-term effects of this early stress on spatial learning and memory, and stress reactivity in early P26-34 and late P45-53 adolescent male and female rats. Intra-plantar injection of formalin induced prolonged and elevated levels of corticosterone in pups and impaired spatial learning and short- and long-term memory in late adolescent males and long-term memory in early adolescent females. There were sex differences in late adolescence in both learning and short-term memory. Performance on the long-term memory task was better than that on the short-term memory task for all early adolescent male and female control and stressed animals. Short-term memory was better in the late age control rats of both sexes and for formalin treated females as compared with the early age rats. These results are consistent with an impaired function of structures involved in memory (the hippocampus, amygdala, prefrontal cortex) after newborn pain. However, activation of the HPA axis by neonatal pain did not directly correlate with spatial learning and memory outcomes and the consequences of neonatal pain remain are likely multi-determined.

## Introduction

The longitudinal analysis of outcomes of pain for premature infants in a neonatal clinic found that pain in these infants resulted in impaired cognitive function in the child and adolescent (Haley et al., 2006; Grunau et al., 2009; Doesburg et al., 2013; Vinall et al., 2014; Chau et al., 2017). Whereas the long-term influence of non-pain stress (e.g., maternal separation, mother and offspring isolation, early handling or the limited nesting model) on cognitive behavior and memory in animal models is abundant in the literature (Krugers and Joëls, 2014; Schroeder et al., 2018; Bonapersona et al., 2019; Cordier et al., 2021), the long-term consequences of pain stress have been understudied (Khawla et al., 2017), especially considering it prevalence in the clinical setting (Ranger and Grunau, 2014; Mooney-Leber and Brummelte, 2017) and the strong connection between neonatal pain and disturbances of central nervous system maturation (Schwaller and Fitzgerald, 2014; Brewer and Baccei, 2020; Williams and Lascelles, 2020). The neonatal period is a critical period of development due to the rapidly changing neurobiological processes, which result in altered sensitivity to external stimuli and a high level of plasticity of the nervous system (Lupien et al., 2009). The nervous system during the neonatal period is highly sensitive to painful stimuli (Goksan et al., 2015; Williams and Lascelles, 2020) with the critical period for later effects on adult pain ending around the end of the first week of life in the rat (Ren et al., 2004). It is known that repeated pain in the neonatal period disrupts the balance between the processes of excitation and inhibition in the central nervous system (Brewer and Baccei, 2020), modifies brain development (Schwaller and Fitzgerald, 2014), programming the HPA axis (Mooney-Leber and Brummelte, 2017), therefore modifying multiple types of behavior (Williams and Lascelles, 2020). Moreover, there is a close neuroanatomical and physiological interaction between pain and the HPA axis, which is regulated in part by the hypothalamus, amygdala, hippocampus, prefrontal cortex (PFC), and thalamus (Ulrich-Lai and Herman, 2009; Victoria et al., 2013; Timmers et al., 2019). The features of this interaction in response to damaging stimuli at an early age are poorly understood. Because there are multiple physiological systems affecting pain and the HPA axis in the neonatal period (Mooney-Leber and Brummelte, 2017, 2020; Van Bodegom et al., 2017) the data on the effect of pain on the HPA axis, both in the clinic and in animals, are incomplete (Walker et al., 2003; Victoria et al., 2013; Victoria and Murphy, 2016). Moreover, the effects of stress and pain on the HPA are age dependent, even in infancy (Van Bodegom et al., 2017). Further research is needed to clarify the relationship between neonatal pain and the HPA axis, since there is a multifaceted relationship between the type of pain, severity, gender, and age of pain exposure and the response of the HPA axis.

In adolescence, problems of neurodevelopment and behavior caused by infant stressful influences first appear. To correct the behavior during the adolescent period, it is important to know the features of the age-related intervals of an adolescent development period. Adolescence is characterized by intensive processes of synaptogenesis and myelinization, especially in the prefrontal cortex, hippocampus and amygdala (Brenhouse and Andersen, 2011), reorganization in the hormonal, neurotransmitter, and reproductive systems (McCormick et al., 2004, 2016; Romeo and McEwen, 2006; Romeo, 2010; McCormick, 2011), and behavioral and cognitive maturation (McCormick and Mathews, 2010; McCormick et al., 2012; Siddiqui and Romeo, 2019). The trajectory of these processes in adolescence can be altered by neonatal stress, including clinically necessary painful procedures (Amaral et al., 2015; Chen et al., 2016; Mooney-Leber and Brummelte, 2017; Xia et al., 2020), which can modify behavior and cognitive abilities in adolescents (Mooney-Leber and Brummelte, 2020; see Williams and Lascelles, 2020 for a review). Thus, early-life pain can give rise to numerous clinical, social, educational problems in adolescents which may differ from those of adults.

Based on behavioral and the nervous system maturation, adolescence in rats ranges from 28-48 days (P28-48) with adults defined as P60 and older (Spear, 2000). A more granular view of this critical period of development divides adolescence into three sub-periods: early P21-P34, middle P34-P46 and late P46-P59 (Tirelli et al., 2003). These epochs within adolescence vary somewhat depending on which criteria are used (morphogenetic, behavioral, neurohormonal, and neural). Much attention has been paid to the different neurobiological systems within the age-related intervals of adolescence (McCormick et al., 2016, 2020; Bailey et al., 2020; Marcolin et al., 2020; Gore-Langton et al., 2021). One of the main factors determining the timing of adolescence is the maturation of the hypothalamic-pituitary-adrenal system (the HPA axis), and its feedback mechanisms, which continue to mature during adolescence. Glucocorticoids, the secretion of which is controlled by the HPA axis, affect brain development, including neurogenesis, synaptogenesis, and cell death (McEwen, 2000). The development of the HPA axis during adolescence may be modified by stress experienced early in life (Van Bodegom et al., 2017). The timing of adolescence can also be determined by sexual maturation and its relationship with the HPA axis (McCormick and Mathews, 2007). The pubertal onset of sexual maturation, determined by vaginal opening in female rats and preputial separation in male rats, occurs earlier in females (P35 ± 2) than in males (P42 ± 2) (McCormick and Mathews, 2007, 2010), making it important to include males and females at different stages of adolescence in studies. The peripheral steroid hormone of the HPA axis, cortisol in humans, corticosterone in rodents, plays an important role in learning and memory (Akirav et al., 2004). The effects of inflammatory pain on the secretion of corticosterone in newborns, and the consequences of these effects on the development of brain structures involved in cognitive function and in the formation of HPA axis before puberty are still largely unknown. Repeated prick needles of the pad of hind paws have been used as a pain stressor in newborn rodents (Anand et al., 1999; Walker et al., 2003; Nuseir et al., 2015, 2017; Ranger et al., 2019); however, the results are often mixed. For example, repeated needle pricks to the pad of the hind paws of newborn rodents did not change the level of corticosterone in adult rats (Anand et al., 1999; Walker et al., 2003), but did decrease stress reactivity of the HPA axis in adolescence, and impaired the ability to retain spatial memory in prepubertal male rats (Chen et al., 2016). In prepubertal mice, similar injury impaired spatial learning and short-term (Nuseir et al., 2015) and long-term (Nuseir et al., 2017) memory, whereas in adult mice, it impaired short-term memory, but did not alter spatial learning ability and long-term memory (Ranger et al., 2019).

We are aware of only a few rodent studies that investigated the effect of neonatal inflammatory pain on memory. For instance, inflammatory pain caused by the intraplantar injection of carrageenan (1%) on the day of birth (P0), resulted in spatial memory deficits in adult rats (Henderson et al., 2015), and also changed the regulation of the HPA axis (Victoria et al., 2013); complete Freund’s adjuvant on P1 did not affect short-or long-term memory in male or female rats on P60, but resulted in spatial learning deficits in males (Amaral et al., 2015). Formalin-induced pain in newborn rats, which produces less prolonged pain than does carrageenan or CFA, impaired visual-spatial learning and memory in the radial 8-arm maze, which uses food reinforcement, in adult rats (Anand et al., 2007).

There are many models of neonatal pain, each with their advantages and disadvantages. All are meant to model the experience of the infant in the NICU who experiences many skin breaking experiences each day (Grunau et al., 2006). The four most common are repeated needle stabs, or carrageenan, CFA or formalin injection, although others exist (e.g., paw incision; local capsaicin treatment). Formalin has the advantage of producing a reliable short-lived behavioral response (<1 h) and accompanying edema (Anand et al., 1999), without long-term immune activation or substantial disruption of mother-pup interactions, therefore limiting the duration of pain and allowing precise control over the age of injury.

We previously showed that inflammatory pain on P1and P2 did not alter spatial learning in 33-day-old adolescent female rats (Butkevich et al., 2020). A more granular study of the consequences of inflammatory painful effects on cognitive abilities and the stress-hormonal system in adolescence is especially important, since neurobiological and behavioral changes vary greatly at different stages of adolescence and these changes, caused by stressful effects at an early age, may be manifested specifically at one or more of these stages. Identifying this unique developmental pattern would help direct efforts to ameliorate any untoward effects of early stress pain more precisely.

Most of the basic research has been and is being done on males (Chen et al., 2016; Xia et al., 2020). When both sexes are tested, females have been found to be more vulnerability to the noxious influences at an early age by some (Gildawie et al., 2021), whereas others report increased vulnerability in males (Van Dammen et al., 2020). Androgens in adults inhibit the activity of the HPA, whereas estrogens, on the contrary, increase it (Handa et al., 1994; McCormick et al., 2002). Pain-related sex differences are present from birth (Verriotis et al., 2018; Gursul et al., 2019). Pain stress is often presented in the neonatal clinic, and it is important to know how consequences of pain stress are manifested in different term intervals of adolescence in order to correct the behavior in this period of postnatal development.

The aim here was to investigate the immediate effect of formalin-induced pain in one-day-old and two-day-old (P1, P2) rat pups on corticosterone level in blood plasma and the long-term effects of early-life pain stress on spatial learning and memory in the Morris water maze, and stress reactivity of the HPA axis in male and female rats of early P26-34 and late P45-53 age groups of adolescence.

## Materials and Methods

### Animals

Subjects were the offspring of Wistar rats (parents: males, *n* = 35 and females *n* = 62) from the biocollection of Pavlov Institute of Physiology of the Russian Academy of Sciences. After two days of adapting to new quarters, the rats were mated, and a vaginal smear was examined next morning to verify insemination. The days of insemination and birth were considered as gestational day (G) 0 and postnatal day (P) 0, respectively. Pregnant dams were housed four per cage, then individually after the 17th day of pregnancy. All animals were maintained under standard conditions (12 h light, 12 h dark, lights on at 08:00, 20–22°C) in standard plastic rat cages with food and water available *ad libitum*. The birth of offspring was checked at 8, 13, 17 and 20 h. A day after the birth of the offspring, litters were reduced to 8 rat pups (4 males and 4 females if possible). From each mother, one male and one female were included in the experiment; in two cases, two rats of each sex from one dam were used. In the latter case, data from these two animals were averaged to produce a “litter” mean (see the statistics section below). The remaining rats in a litter were used in other experiments. All procedures were approved by the Local Ethics Committee for Animal Experiments of the I. P. Pavlov Institute of Physiology, Russian Academy of Sciences (Saint Petersburg, Russia) and followed the guidelines published by the Committee for Research and Ethical Issues of the IASP on ethical standards for investigations of experimental pain in animals.

### Neonatal Inflammatory Pain

On the first and second day of life (P1 and P2) offspring of both sexes were injected with the inflammatory agent formalin (2.5%, 0.5 μl) into the pad of the left hind paw; as a control, a single prick needle or saline injection was used. In preliminary experiments, in which P1 and P2 rat pups were subjected to a single needle prick (*n* = 5 in each adolescent age and sex group) or a single injection of saline solution into the pad of the left hind paw (*n* = 5 in each adolescent age and sex group), the animals were tested at P26-34 and P45-53. No differences were evident in spatial learning and memory in the Morris water maze (MWM) or in the stress reactivity of corticosterone between the pricked and saline animals in both age and sex groups (see Supplementary Figures 1–4), so needle prick rats were used as a control for formalin injection in the MWM experiments and saline injection as control for the corticosterone assays in newborn rat pups. In addition, the rats designed to determine basal corticosterone levels were handled at the same time as Control or Formalin rats but otherwise untreated. P1 and P2 in rats roughly correspond to extreme prematurity in human (gestation weeks 24), based on development of the brain and pain system in rats and humans (Dobbing, 1981; Fitzgerald et al., 1988). All animals in each litter were randomized to the inflammatory pain (Formalin) and Control. Formalin rats were labeled with a weak solution of picric acid along the back, control rats remained unmarked; remaining rats designed for other experiments, had their heads painted with picric acid.

### Morris Water Maze (MWM)

A modified version of the MWM was used to assess spatial learning, spatial short-term and long-term memory (Morris, 1981; Vorhees and Williams, 2014). The MWM consisted of a round tank (120 cm diameter, 72 cm deep) filled to 40 cm with opaque with chalk-clouded water (24 ± 2°C) to eliminate the platform’s visibility. The tank, located in a room with several strongly contrasting extra maze cues, was visually divided into four equal quadrants West (W), South (S), East (E) and North (N). A steel platform (39 cm height, 12 cm diameter) was placed in the SW quadrant approximately 40 cm from the side wall, and its location fixed for all the animals during all training days. The investigator was visible to the rats, and her location was constant throughout all experiments. The installation was illuminated by two lamps (250 W), the light from which was directed to the ceiling to obtain soft diffused lighting.

### Spatial Learning Assessment

Spatial learning tests were conducted at two age groups during the adolescent period: early (P26-34) (formalin rats *n* = 15 males and *n* = 12 females, and control rats *n* = 16 males and *n* = 14 females) and late (P45-53) (Formalin rats *n* = 15 males and *n* = 15 females, and Control rats *n* = 16 males and *n* = 16 females). Rats tested at the early age were with their mother until the end of the experiments (on the 34th day of life). The rats of the late age group were weaned also at 34 days, and males and females placed in different cages, 3–4 per cage. Both cohorts were tested identically.

In the MWM, each rat was trained for 5 consecutive days to locate a platform with eight training trials per day, divided into four trials with an interval between them of 4 minutes. For each training trial, the rat was placed in the water facing the tank wall, in the first training trial into the NW quadrant, then consistently in the SW, SE and NE quadrants. The rat was allowed to search for the platform for 60 s. Failing that, the experimenter placed the rat on a platform, where it remained for 20 s to learn its location relative to the extra-maze visual cues. Then the rat was transferred to a dry cage with paper towels as bedding for 15 s, after which the training trials were continued. The latency (from when the rat was submerged in the water tank to when it located the platform) was recorded in each trial. If the rat did not locate the platform during a trial, it was assigned the maximum trial duration 60 s as the score for that trial. The average latency in the first four training trials and the average latency in second four training trials were used as measures of learning.

In addition to latency, we used the index of acquisition and the savings index as additional measures of spatial learning during training tests (Whiting and Kokiko-Cochran, 2016; Tucker et al., 2018). The index of acquisition describes the learning that occurs within one day of testing, and is calculated by taking the difference between the latency in the first and last tests and averaging this difference for all days of spatial learning. The savings index is the measure of how well, on the first test of each day, the rats remember what was learned on the previous day. This value is calculated as the difference between the latency in the last test of a given day and the latency in the first test of the next day and averaged over all days of spatial learning. Thus, the savings index reflects consolidating and storing memory and/or its retrieval process.

### Spatial Memory Assessment

On the fifth and last training day, the rats were exposed only to the first four training trials, and then dried and returned to their cages in a different room. After one hour, each rat was placed back into the pool and the spatial short-term memory was examined without the platform. This short-term assessment reflects a combination of reference and working memory (Vorhees and Williams, 2014). The NW quadrant start location was used for short- and long-term memory assessment. Long-term spatial memory, memory retention, was examined 96 h after the short-term memory study (34th and 53d days of life in the early and late age groups), by placing each animal sequentially in water without a platform. Animals were allowed to swim freely for 60 s in the water tank without the platform. The latency to locate the spot where the hidden platform had been previously and the amount of time the animal spent in the target quadrant (SW quadrant) were the scores for the short- and long-term memory probe trials. Behaviors in the short- and long-term memory tests were recorded using a webcam with automatic focusing (Microsoft 5WH-00002) and also visually in real time.

### Blood Collection and Corticosterone Determination

There were 56 newborn rat pups (basal, *n* = 19; saline (control) *n* = 18; formalin, *n* = 19) used for determination of corticosterone in the blood. Blood samples were collected by rapid decapitation without anesthesia. Basal samples were taken at 9 AM. To determine the effect of inflammatory pain in newborn rats on the activity of the HPA axis, blood samples were collected following decapitation of one female and one male into a single test tube (the volume of blood from one rat neonate is very small, so we combined samples from one male and one female in one test tube) 30 minutes after subcutaneous injection of formalin (2.5%, 0.5 μl) or saline into the pad of the left hind paw. The time course of the effects of the formalin treatment on corticosterone levels were evaluated one day and seven days after injection of formalin or saline. In early adolescent rats (basal, *n* = 8 males and *n* = 8 females; control (needle prick), *n* = 10 males and *n* = 9 females; formalin, *n* = 10 males and *n* = 8 females) and late adolescent rats (basal, *n* = 7 males and *n* = 11 females; control (needle prick), *n* = 6 males and *n* = 6 females; formalin, *n* = 6 males and *n* = 6 females), corticosterone reactivity to the forcing swimming was determined 30 min after the long-term spatial memory test in MWM. Here, unlike the corticosterone assay in newborn rat pups, blood of males and females was collected in separate test tubes by rapid decapitation without anesthesia. Following collection, blood was centrifuged, and blood plasma was stored in a freezer (−20°C). Corticosterone was determined in duplicate by immune-enzyme analysis, using standard kits (“Xema-Medica Co” Cat No: K210R; Russia); the intra-assay coefficient was 3.8.

### Statistical Analysis

Mixed ANOVA was used for spatial learning Analysis I for the first four training trials and Analysis II for the second four training trials, the within-subjects factor was day of testing (days 1,2,3,4,5), and between-subjects factors were age (26–30 days/45–49 days), sex (males/females), exposure (formalin P1 & P2 or needle prick at P1 & P2); when Mauchly’s Test of Sphericity was significant, we used Greenhouse-Geisser method. A mixed ANOVA was used for comparison of the first four and second four training trials, Analysis III, the within-subjects factors for day number (1,2,3,4 on each of four days), between-subjects factors were the same as Analyses I and II. For the index of acquisition, three-way analysis of variance ANOVA was used; the factors were age, sex, exposure. For the savings index, three-way (factors: age, sex, exposure) and two-way (factors: age, exposure) analyses of variance ANOVA were used. For memory, mixed ANOVA was used, within-subjects factors: memory (short-time memory, STM/long-term memory LTM) for latency (time to find platform location) and for time the animal spent in the target quadrant, and between-subjects factors age (30 and 34 days for STM and 49 and 53 days for LTM), sex (males/females) and exposure (formalin P1 & P2/needle prick P1 & P2). For corticosterone in newborns, a one-dimensional two-factor analysis of variance ANOVA was used. The dependent variables were corticosterone, day of test (30 minutes, first day, seventh day) and exposure (basal level, saline, formalin); for corticosterone of adolescent rats, three-way univariate analysis of variance ANOVA was used, and the between factors were day of test (34 days/53days), sex (males/females), exposure (basal/needle prick/formalin). Comparison of corticosterone separately for males and females did not reveal sex differences; therefore, a variant of paired comparisons was made for males and females in total. *Post hoc* comparisons were made with Bonferroni multiple comparisons test. For two cases, when two rats of each sex from one dam were used, we averaged the data from littermates (where they were 2 from a litter) to create a single data point per litter. We ran the analysis of variance for litters per group, subjected to the same exposure. The analysis showed that in the majority of the groups of the rats there is no principal differences between the results of dimension in different litters, this indicates that the litters are uniform for the most part and we re-ran the statistical analysis this way.

## Results

Details of the analyses of main experimental data are in Table 1 and summarized below.

**TABLE 1:** Details of the statistical analyses.

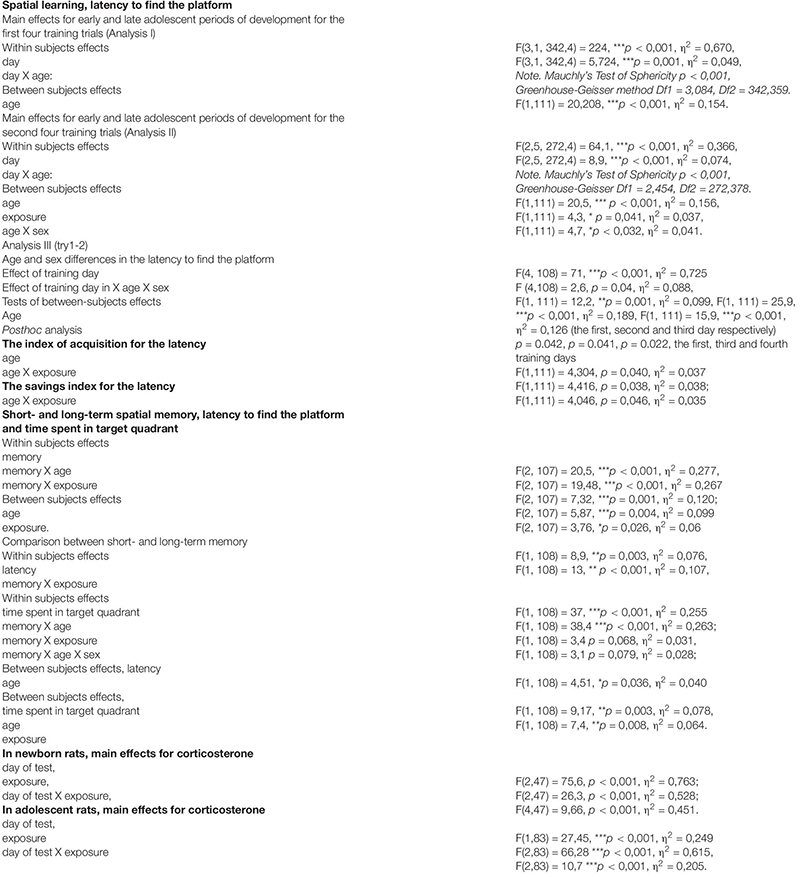

### Spatial Learning, Latency to Find the Platform

**FIGURE 1:**
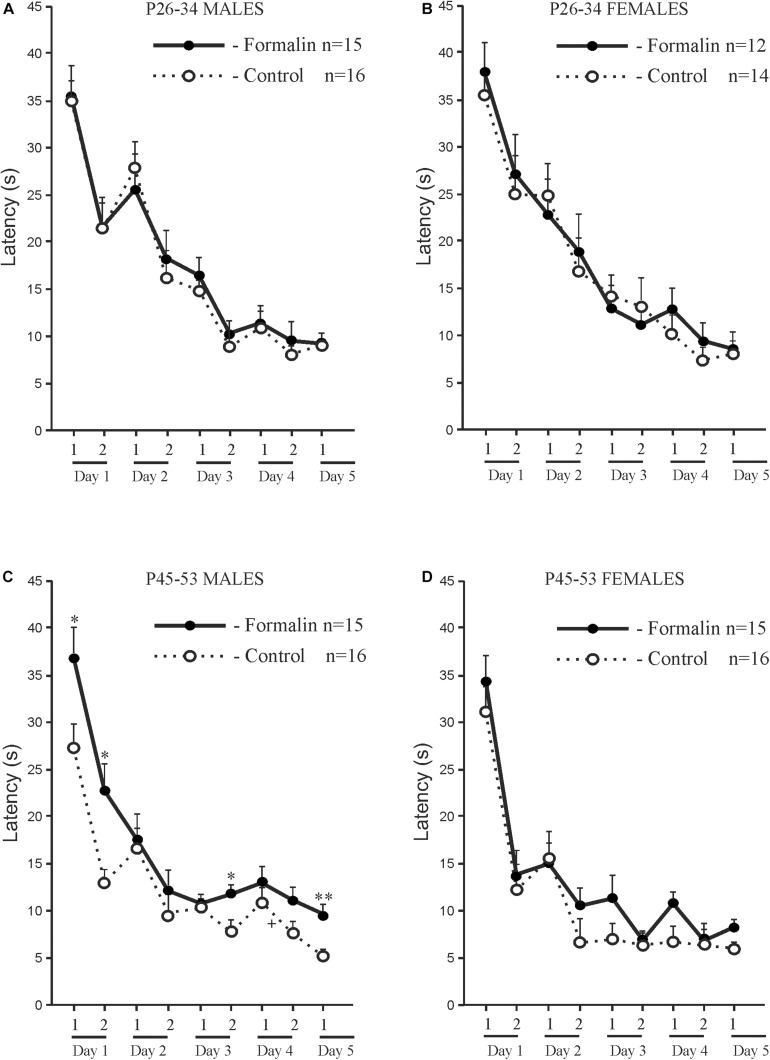
Mean (±SEM) latency to find the platform in the first four training trials for 5 days and second four training trials for four training days of spatial learning in Control and Formalin male and female rats of early (P26-34) and late (P45-53) age groups. Panels **(A,B)** show data for male and female rats at the early age group. Panels **(C,D)** show data for male and female rats at the late age group. The abscissa shows the first and second training four trials (1 and 2) in each of the five training days. ^+^*p* < 0.05, ^++^*p* < 0.01, ^+++^*p* < 0.001 significant differences in Control rats between the first four training trials and the second four training trials each day. ^∗^*p* < 0.05, ^∗∗^*p* < 0.01 Formalin rats *vs* Control rats.

Both Control and Formalin rats in both age groups and of both sexes showed spatial learning, since the latency to find the platform decreased on the fifth training day in all the animals (Figure 1). However, there were differences in spatial learning between P26-P34 and P45-P53 rats. For early adolescent period (P26-P34) (Figures 1A,B) *post hoc* analysis found no differences in the latency to find the platform between the Control rats (males *n* = 16, females *n* = 14) and Formalin rats (males *n* = 15, females *n* = 12) in either training trial on each of the 5 training days. Neonatal formalin pain did not alter the latency to find the platform. For late adolescent period (P45-P53) (Figures 1C,D) *post hoc* analysis found that neonatal pain significantly increased the latency to find the platform in Formalin males (*n* = 15) compared to the latency in Control males (*n* = 16) (Figure 1C).

**FIGURE 2:**
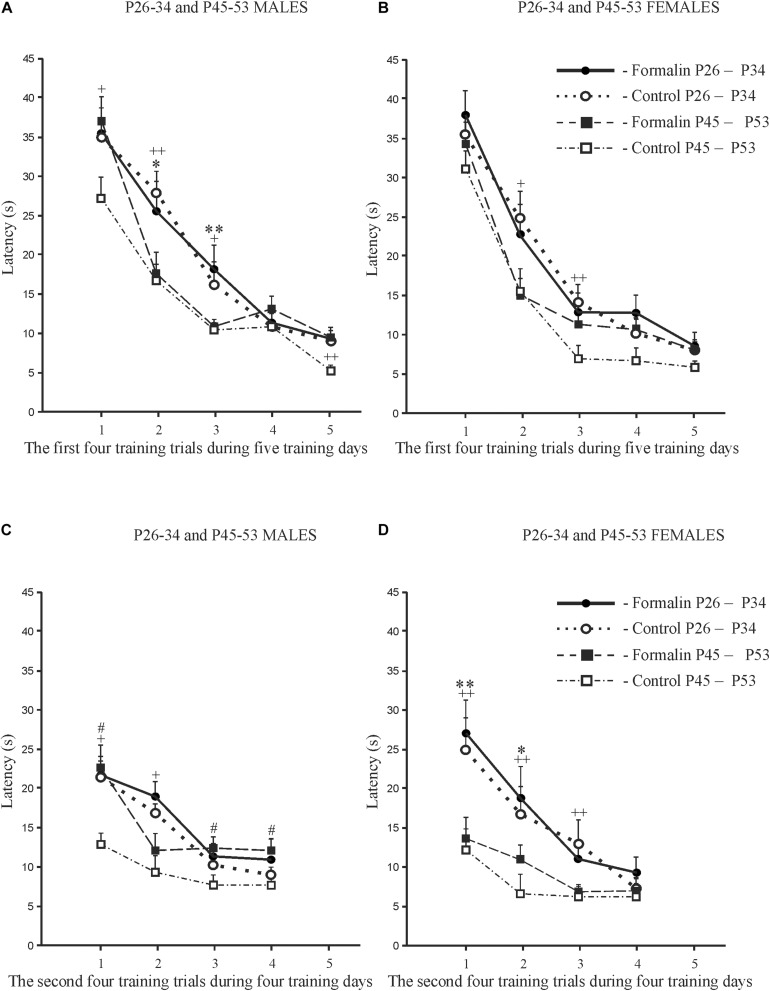
Age and Sex effects in the mean (±SEM) latency to find the platform in Control and Formalin male and female rats in the first four training trials during five training days [early P26-34 and late P45-53 age groups **(A,B)**] and in the second four training trials during four training days [early P26-34 and late P45-53 age groups **(C,D)**]. ^∗^*p* < 0.05, ^∗∗^*p* < 0.01 age differences in Formalin rats, ^+^*p* < 0.05, ^++^*p* < 0.01 age differences in Control rats. ^#^*p* < 0.05 sex differences in P45-P48 Formalin rats. Abscissa, training days. The number of the rats in the groups corresponds to the number of rats in Figure 1.

Age and sex differences in the latency to find the platform are presented in Figure 2. The latency was longer in P26-34 rats of both sexes. Age differences were found in Control males (Figures 2A,C) and Control females (Figures 2B,D) in the first four (Figures 2A,B) and second four (Figures 2C,D) training trials. It was found, that in the first training day, that characterizes the greatest response to the stimulus in MWM (Vorhees and Williams, 2014), neonatal pain significantly increased the latency to find the platform in P45-53 males in both four training trials to the levels of the younger males, whereas in females, similar effects of formalin were not evident. The greater latency to find the platform in the late adolescent period in Formalin P45-53 males compared to the latency in Formalin P45-53 females indicates their greater vulnerability to the effects of neonatal inflammatory pain on spatial learning in males than in females (Figure 2C).

### The Index of Acquisition (Figure 3A) and the Savings Index (Figure 3B) for the Latency

**FIGURE 3:**
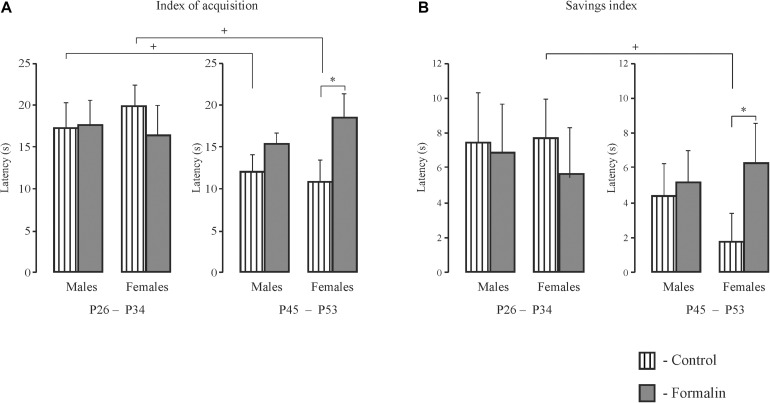
The index of acquisition **(A)** and the savings index **(B)** for latency to find the platform during spatial learning in male and female rats of early (P26-34) and late (P45-53) age groups. The data are: Mean (±SEM) latency differences between the first and last training trials of each of the five training days of spatial learning **(A)**. Mean (±SEM) latency differences between the last training trial of a given day and the first training trial of the next day during the five-day spatial learning **(B)**. Both indices illustrate a decrease of latency to find the platform with age in Control rats; neonatal formalin-induced pain leveled the age differences. ^∗^*p* < 0.05 Formalin *vs* Control **(A,B)**; ^+^*p* < 0.05 Control P45-49 rats *vs* Control P26-34 rats **(A,B)**. The number of the rats in the groups corresponds to the number of rats in Figures 1, 2.

We used the index of acquisition and the savings index as supplemental ways to assess the effectiveness of spatial learning. The index of acquisition (Figure 3A) and the savings index (Figure 3B) support the data presented in Figures 1, 2, indicating that the spatial learning within a day was more successful (latency was shorter) in Control P45-53 rats than in Control P25-34 rats (*p* < 0.05, males and females). The savings index (Figure 3B) demonstrated that Control P45-53 females, compared to Control P25-34 females, (*p* < 0.05), recalled better on the first trial of each day what was learned on the last trial of the previous day. Neonatal formalin pain neutralized these age-related differences. In the early age group of rats of both sexes, neither index showed effects in the formalin treated animals. Indeed, the index of acquisition and the savings index indicated that formalin treated P45-53 females showed deficits in the both learning and memory (the latency was greater) compared to Control females of the same age. These data are not consistent with those data in Figure 1. It is likely that the scoring mechanism underlying this alternative method determines these differences, as reflected in the sex differences in the first four and second four training trials.

### Short- and Long-Term Spatial Memory, Latency to Find the Platform (Figure 4)

**FIGURE 4:**
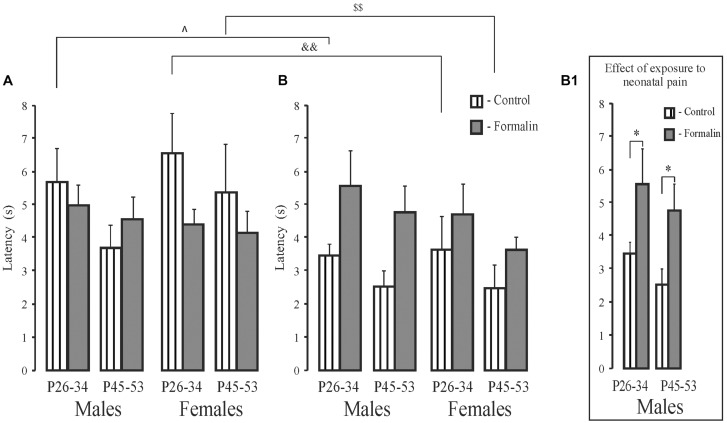
Mean (±SEM) latency to find the platform **(A,B)** for short-term **(A)** and long-term **(B)** spatial memory in the Formalin or Control male and female rats for the early (P26-34) and late (P45-53) age groups. Differences in latency between short-term and long-term memory were found in the Control male and female rats of the early age group and in females of the late age group. Formalin *vs* Control rats; differences between short- and long-term memory: in latency, ^∧^*p* < 0.05, ^&⁣&^*p* < 0.01, in Control P26-34 males and females, and ^$$^*p* < 0.001, in Control P45-53 females. The number of the rats in the groups corresponds to the number of rats in the groups in Figures 1, 2. The graphs on the right illustrate significant results of statistical analysis. ^∗^*p* < 0.05 significant effect of exposure.

In latency to the target quadrant, there were no age or sex differences in the treated groups for either the short- and long-term memory measures. However, the formalin treated males, but not females, showed long-term memory deficits at both ages (Figures 4B,B1) with no differences in the short-term memory task (Figure 4A). Control P26-34 rats of both sexes found the quadrant where the platform had been previously more quickly in the long-term compared to the short-term memory task (*p* = 0.018 and *p* = 0.006, males and females respectively) as did P45-53 females (*p* = 0.003) (Figures 4A,B). Thus, neonatal pain impaired long-, but not short-term memory, in the males, but not females, in both ages. Formalin pain neutralized the differences in latency between short-term and long-term memory, which were found in Control animals.

### Short- and Long-Term Spatial Memory, the Time Spent in Target Quadrant (Figure 5)

**FIGURE 5:**
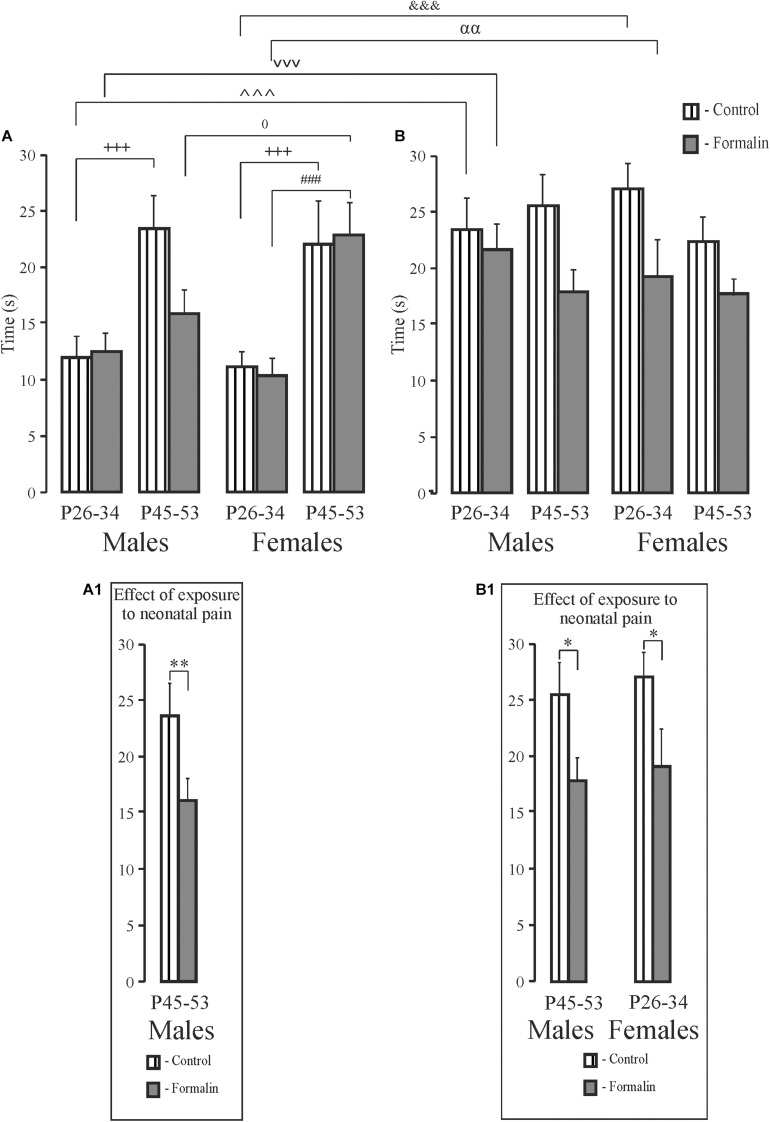
Mean (±SEM) time in target quadrant **(A,B)** for short-term **(A)** and long-term **(B)** spatial memory in the Formalin or Control male and female rats for the early (P26-34) and late (P45-53) age groups. Differences in time in target quadrant between short-term and long-term memory were found in Control and Formalin males and females of early age groups. In all cases, the time in target quadrant was shorter in the short-term memory **(A,B)**. ^+++^*p* < 0.001, age differences in Control rats; ^###^*p* < 0.001, age differences in Formalin rats. Differences between short- and long-term memory: in time in target quadrant, ^∧∧∧^*p* < 0.001, ^&⁣&⁣&^*p* < 0.001 in Control P26-34 males and females, ^vvv^*p* < 0.001, ^αα^ *p* < 0.01, in Formalin P26-34 males and females; ^0^*p* < 0.05, sex differences in P45-53 Formalin rats. The number of the rats in the groups corresponds to the number of rats in Figures 1, 2. The graphs below illustrate significant results of statistical analysis. ^∗^*p* < 0.05, ^∗∗^*p* < 0.01 significant effect of exposure. Graphs **(A1)** and **(B1)** illustrate the significant outcomes of the statistical analyses.

For the time in the target quadrant, Formalin P45-53 male rats spent less time in target quadrant in the short-term memory task than did the same-age Control males (Figures 5A,A1). There were age differences in the short-term memory task in Control males and females and Formalin females (Figure 5A). In the long-term memory task, less time was spent in target quadrant in Formalin P45-53 males and Formalin P26-34 females as compared to the time in Control rats of the same ages (Figures 5B,B1). There were differences between short-and term-long memory performance in P26-34 animals, in both Control and Formalin rats of both sexes (Figures 5A,B). The time spent in the target quadrant was less in the short-term memory than in the long-term memory task. Sex differences were found in the short-term memory test since females spent more the time in the target quadrant in Formalin P45-53 rats than did males (Figure 5A).

### Corticosterone Determination in Newborn Rats (Figure 6) and Adolescent Rats (Figure 7)

**FIGURE 6:**
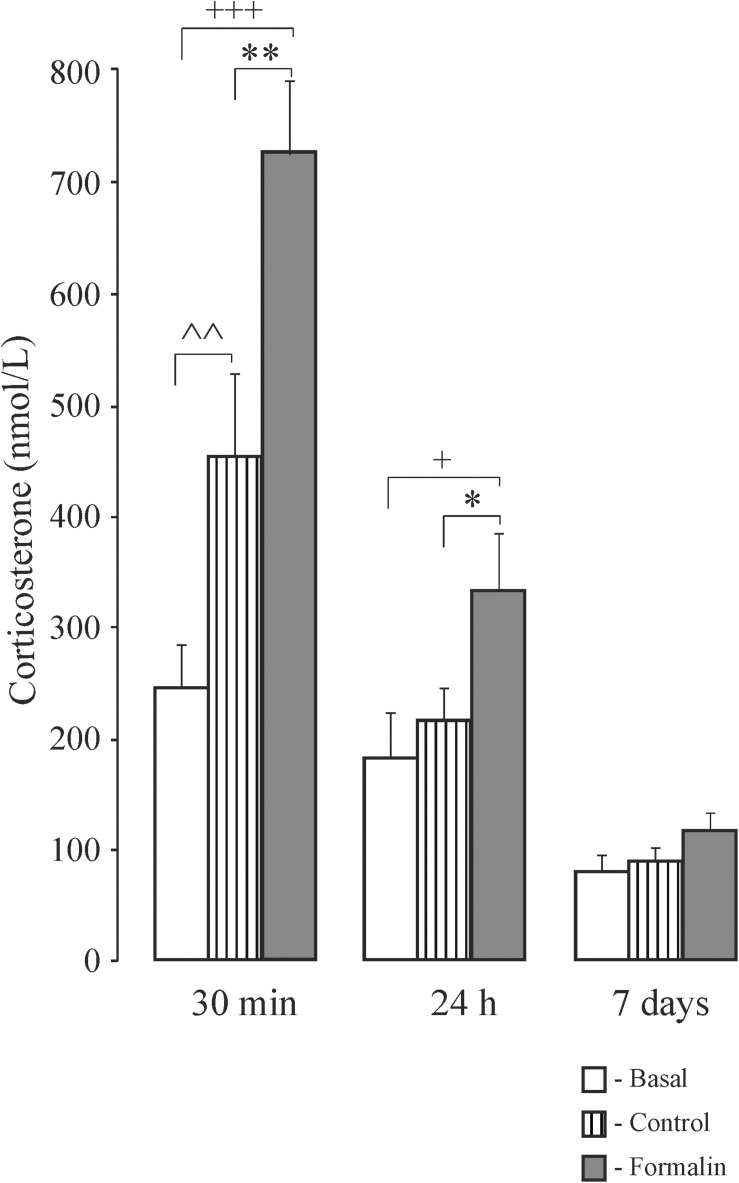
Mean (±SEM) corticosterone levels in blood plasma in neonatal pups under basal conditions, or 30 min, 24 h and 7 days after injection of Formalin (2.5%, 0.5 μl) or Control into the pad of the left hind paw. ^∗^*p* < 0.05, ^∗∗^*p* < 0.01, Formalin *vs* saline; ^+^*p* < 0.05, ^+++^*p* < 0.001, Formalin *vs* basal; ^∧∧^*p* < 0.01, saline *vs* basal.

In newborn rats, 30 min after formalin injection corticosterone levels were higher in the Control and Formalin pups compared to basal levels, and higher in the Formalin pups than in Control pups (Figure 6). Likewise, twenty-four hours after formalin injection, the Formalin pups had a higher corticosterone level than the Control and the basal pups. Seven days after formalin injection, there were no significant differences in the corticosterone levels among the three groups of pups.

**FIGURE 7:**
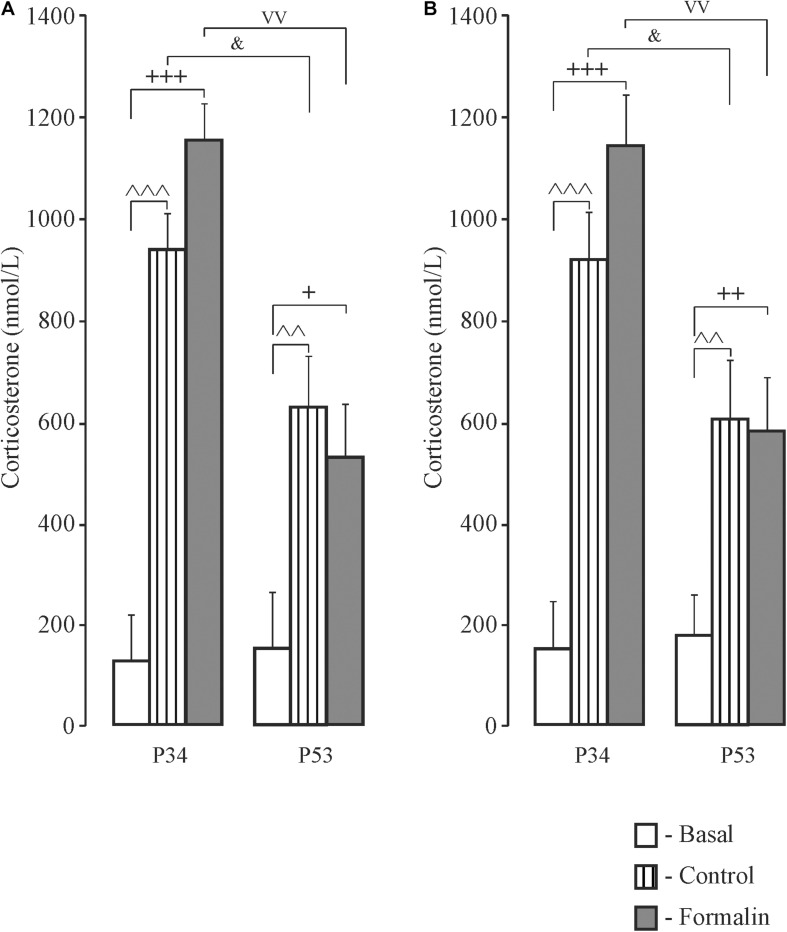
Mean (±SEM) corticosterone levels in blood plasma in response to forced swimming in Control and Formalin rats at the age of P34 and P53 after testing in the Morris Water Maze. ^+^*p* < 0.05, ^++^*p* < 0.01, ^+++^*p* < 0.001, Formalin *vs* basal; ^∧∧^*p* < 0.01, ^∧∧∧^*p* < 0.001 Control *vs* basal; ^&^*p* < 0.05, age differences between Control rats; ^vv^*p* < 0.01, age differences between Formalin rats. The number of the rats in the groups corresponds to the number of rats in Figures 1, 2.

In adolescent rats, there were no sex differences in corticosterone levels and therefore male and female data were combined. Thirty min after forced swimming, which rats were subjected to after long-term memory testing, corticosterone levels were higher compared to basal levels in both the Control and Formalin groups in early and late ages (Figure 7). Age differences were found in both sexes in the Control and Formalin rats with greater corticosterone levels in early age group.

## Discussion

The goal of this work was to examine the acute and subacute effects of an injury to the pad of the hind paw on plasma corticosterone, a marker of stress reactivity, in newborn rat pups. Corticosterone was elevated quickly and that elevation was maintained for at least 24 h compared to basal levels and saline injection controls. This suggests that neonatal inflammatory pain could modify the development of the HPA. We hypothesized that this could lead to changes in stress reactivity and cognitive abilities in adolescent rats. Indeed, there were differences in adolescent rats’ plasma corticosterone in response to a swim stress and in aspects of spatial learning and memory depending on whether early and late age rats were tested. In the early P26-34 and late P45-53 age groups, the effects of repetitive neonatal peripheral inflammatory pain on spatial learning, short-term and long-term memory, strongly indicate a pronounced heterogeneity of the effects of early pain during the adolescent period of rat development. However, the long-term effects of early formalin injury and subsequent HPA activation cannot explain the adolescent effects in any simple way.

In neonates, the HPA axis rapidly develops and responds to strong stressors (Wood and Walker, 2015). We had previously shown that even during the stress hyporesponsive period of the HPA axis development, formalin-induced pain caused a gradual over an hour an increase in plasma corticosterone levels in 7-day-old male rats; twenty-four hours after injection of formalin corticosterone still exceeded the basal value (Butkevich et al., 2013). We also showed that neonatal formalin-induced pain caused an increase in an inflammatory pain response, depression-like behavior, and impairment of learning in adolescent male rats (Butkevich et al., 2016). But the question remains open as to whether the altered endogenous cortisol following early pain stress may play a role in learning and memory performance, as has been suggested in children (Mooney-Leber and Brummelte, 2017), especially in the prepubertal period in both sexes.

Weaning is stressful for the offspring. In the present work, weaning occurred on P34 in both age groups. Experiments with rats of the early group were conducted before weaning, the pups after the experiment were in the home nest with their mother, while the pups of the late group were without the mother, but with their sisters or brothers. If weaning took place in P25, as is usual in our laboratory (Butkevich et al., 2017), then testing with P26 would be more stressful for the early group of rats than testing with P45 for the late group of rats. So, the rats had weaning at a later age. The rats of the late age group had enough time (11 days) for adaptation of life without the mother. In the MWM, the early age Control rats were capable of spatial learning, which is consistent with the literature (Vorhees and Williams, 2014), but which is in contrast to earlier work that found that effective strategies for spatial learning in the Morris water maze appear relatively late in adolescence (P42) (Schenk, 1985). Probably, the differences in the line of rats (Wistar and Hooded rats) and the testing methodology (in Schenk, 1985, Hooded rats were allowed to swim only for 30 s) underlie these differences. Formalin treated males and females of both age groups also demonstrated spatial learning, as evidenced by the gradual decrease in the latency of finding the platform during five training days. However, compared to controls, older male but not younger male formalin-induced neonatal pain rats took longer to find the platform for both four training trials. This difference (35%) was particularly pronounced on the first training day and it is first training day that is an important criterion for the learning process (Vorhees and Williams, 2014). In contrast to males, females of neither age group showed differences between Formalin and Controls on the first training day. Differences in spatial learning between age groups in Control and Formalin females only appeared in the second four trials. These sex differences may be due to different rates of adolescent sexual maturation which occurs later in males (∼P42 ± 2), than in females (∼P35 ± 2) (McCormick and Mathews, 2007, 2010). This suggests that sex hormones can be one of the reasons for these differences between males and females. Another reason for these differences may be the different reactivity of the HPA axis in males and females. However, no differences in corticosterone reactivity were found between sexes after assessing long-term memory. Interestingly, when using other metrics for spatial learning, the index of acquisition and the savings index (Whiting and Kokiko-Cochran, 2016), we found an increase in the latency to find the platform, which is impairment of cognition, in Formalin P45-53 females, as compared to Control P45-53 females, but not in males of the same age group. The two different metrics measuring learning and memory can explain this difference. The index of acquisition – measure of the learning within one day of testing, and is calculated by taking the difference between the latency in the first and last tests and averaging this difference for all days of learning. The savings index is the measure of how well, on the first test of each day, the rats remember what was learned on the previous day. This value is calculated as the difference between the latency in the last test of a given day and the latency in the first test of the next day and averaged over all days of learning. However, the absence of differences in the latency to find the platform between Formalin and Control rats in the early P26-34 age group was the same as the results obtained using these indices and analysis I and II.

When assessing memory by the latency to find the platform, neonatal pain caused deficits only in long-term memory in males in both age groups, whereas when assessing memory by the time spent in the target quadrant, neonatal pain decreased it in males of the late age group in both short- and long-term memory and also in females of early age group. Only control rats of both sexes of the early age group showed differences between short-and long-term memory in both latency and time spent in the target quadrant, with shorter latency and longer time spent in the target quadrant in long-term memory. In the time spent in the target quadrant, Formalin rats of the early age group, showed the similar behavior. Note, age differences were found only in short-term memory in Control rats of both sexes and Formalin females, and only in the target quadrant, with a longer parameter in the late age group. Differences identified in memory processes using latency to find the platform and the duration to stay in the target quadrant indicate participation of different brain structures in these behavioral characteristics of memory.

We are aware of only a few rodent studies that investigated the effect of neonatal inflammatory pain on memory. For instance, formalin-induced pain in newborn rats impaired visual-spatial learning and memory in the radial 8-arm maze, which uses food reinforcement, in adult rats (Anand et al., 2007). Inflammatory pain caused by the intra-plantar injection of carrageenan (1%) on the day of birth, P0, resulted in spatial memory deficits also in adult rats (Henderson et al., 2015), and dysregulated the HPA axis (Victoria et al., 2013). Complete Freund’s adjuvant on P1 did not affect short-or long-term memory in male or female rats on P60, but resulted in spatial learning deficits in males (Amaral et al., 2015). Therefore, although there are some inconsistencies, in general early experiences of painful injury can disrupt adult spatial learning/memory processes. When assessed, the single injection of carrageenan on the day of birth activated the infant HPA axis in rat pups (Victoria et al., 2014). Daily needle pricks in each paw at 6-h intervals until P7 (Chen et al., 2016), decreased serum corticosterone in P24, had no effect at P45 and increased corticosterone in adult rats. Thus, these early insults can have long-term effects on subsequent HPA axis function. However, we know of no comparable data for testing the effects of early inflammatory injury in adolescence.

We measured HPA reactivity in response to forced swimming in the rats after testing in the MWM, and found no differences in corticosterone levels in adolescent rats between Formalin and Control rats of either sex. Importantly, both Control and Formalin rats at the early age showed greater corticosterone levels compared to those of the late age group. The forced swim test is known to stimulate the HPA activity in rats (Mathews et al., 2008), and HPA axis reactivity is modified by previous stress history, especially during critical periods of rapid brain development (reviewed in Meaney and Szyf, 2005). Stress at an early age changes adaptive behavior. For example, we have previously shown that the formalin test preceding the forced swim test sharply reduced the immobility time only in 7-day-old rat pups that had been prenatally stressed but not control pups (Mikhailenko et al., 2010). Our long-term experience with the forced swim test indicates that the severe physical and emotional stress experienced by the rat in this test can obviate the effects of other varied types of stress. It is important to note, that the absence of differences in the reactivity of the HPA axis between Formalin and Control rats in our current study could be a consequence of the cumulative effects of testing in MWM and forced swimming on the activity of the HPA axis. The interaction of different types of stress, especially during critical periods of development, can lead to unexpected results (Sokołowski et al., 2020). Especially interesting and of practical importance is the consequence of suppressing the adverse effects of one stress by another adverse stress (Van Bodegom et al., 2017). Our data using the formalin pain stress in newborns showed suppression of the expected pronociceptive effect of prenatal stress in the formalin test in adolescent rats, but did not reduce depressive-like behavior (Butkevich et al., 2020).

Our present experiments have shown that the activation of the HPA axis by neonatal pain has no direct relationship with spatial learning and memory in rats in adolescence. Other physiological systems besides the HPA axis may be involved in the effects of inflammatory pain in newborns, such as the immune system, which responds to inflammation and stress and can affect brain neurons and cognitive function. The immune system closely interacts with the HPA axis (Gaillard, 2003). Sex differences in microglia, neuroimmune cells, begin to emerge during the prenatal organizational period for sexual differentiation of the brain (Schwarz et al., 2012). Immunocompetent cells of the brain express steroid hormone receptors and are regulated by hormones and activation of the immune system is determined by sex hormones (Lombardo et al., 2021). Moreover, the immune system acts as a regulator of sex differences in brain development and behavior (Nelson and Lenz, 2017; VanRyzin et al., 2018). The immune and sexual systems interact with the HPA axis (Bereshchenko et al., 2018). One can suggest that the sex differences reported here following neonatal pain depend on the balance in maturation of the HPA and the hypothalamus-pituitary-gonadal (the HPG) axis. Neonatal pain, by disrupting the processes of inhibition or excitation in the central nervous system, could modify the synchronization of development of the HPA and HPG systems, which closely interact and affect the neuroplasticity of learning and memory. The hippocampus, medial prefrontal cortex, and amygdala, brain structures implicated in the control of the HPA axis (Herman et al., 2003) and cognition (Euston et al., 2012; Méndez-Couz et al., 2014), mature rapidly during adolescence (Spear, 2000) and can influence sensitivity of the HPA axis to sex hormones and alter cognitive abilities.

The relatively long-lasting high level of corticosterone evoked by formalin-induced pain in newborn rat could impair the development of the PVN. In the newborn rat, the PVN and CA1 of the hippocampus contain GR mRNA expression (Pryce, 2008). The CRH hippocampal system regulates neurogenesis in the hippocampus which is involved in spatial learning and memory (Koutmani et al., 2019). Elevated levels of glucocorticoids have also been shown to impair working and reference memory (Stylianakis et al., 2018). CRH neurosecretory systems release glutamate, in addition to neuropeptides, into the pericapillary space of hypophysial portal vessels, and there is expression of the mRNA for vesicular glutamate transporter-2 in the rat CRH neurons in the PVH (Hrabovszky et al., 2005). Glutamatergic neurons are one of the main links in the processes of learning and memorization (review, Mooney-Leber and Brummelte, 2017). Excessive levels of glucocorticoids enhance the release of glutamate, causing neurotoxicity, which enhances apoptosis, as shown in the hippocampus and other brain regions during the first postnatal week in the rat (Lu et al., 2003; Dührsen et al., 2013). The role of glutamate during development has been primarily associated with the NMDA receptor, which is present at P0 in the rats (Behuet et al., 2019). During normal early development when the NMDA receptor containing the NR2B subunit in the hippocampus of the newborn rat is activated, the corresponding channel remains in the open position much longer than in the mature receptor. In addition, neurons with such receptors develop long-term potentiation, a form of activity-dependent synaptic strengthening, more quickly, which contributes to memory strengthening. The selective loss of NR2B protein and subsequent synaptic dysfunction weakens prelimbic PFC function during development and may underlie early cognitive impairments (Gulchina et al., 2017). We hypothesize that the impairment of the NR2B subunit caused by increased corticosterone in rats with neonatal pain may be associated with the abnormalities in spatial memory that we found.

Short and long exposures to corticosterone differentially tune NMDAR signaling in hippocampus by altering the expression and synaptic presence of NMDAR subunits, allowing adaptations of glutamate synapses (Mikasova et al., 2017). Taking into account the different roles of metabotropic and inotropic glutamatergic and GABAergic receptors in the effect of stress on learning and memory, as well as the mechanism of co-transmission of glutamate with GABAergic neurons (Trudeau and Mestikawy, 2018), it is possible that these complex relationships are involved both in the differences we found in the effect of early pain stress on cognitive abilities in adolescent rats, and in the absence of differences in the reactivity of the HPA axis to stress in the adolescent Formalin and Control rats. It is known that the serotonergic, the HPA axis, glutamatergic, and GABAergic systems are all involved in nociception and are affected by stress (Goudet et al., 2009; Quintero et al., 2011; Bannister et al., 2017; Hernández-Vázquez et al., 2019). Formalin-induced neonatal pain effects various neurotransmitter systems, disrupts the balance between excitation and inhibition in the central nervous system, modifies the development of functional activity of the HPA axis, and thus affects the neurophysiological mechanisms underlying cognitive processes.

In conclusion, we found that activation of the HPA axis by neonatal pain did not directly correlate with spatial learning and memory in adolescence, and therefore the consequences of newborn pain remain are likely multi-determined. Neonatal pain impaired spatial learning and long- and short-term memory in late adolescent males and long-term memory in early adolescent females. The comparative analysis of the memory scores revealed that long-term memory performance was more robust than short-term memory. The differences found in spatial memory performance in MWM in P25-34 and P45-53 rats provide strong evidence of the heterogeneity in the development of cognitive processes in the two age groups of the adolescence. These behavioral changes suggest that neonatal pain causes changes in various structures and neurotransmitters involved in spatial short-term and long-term memory only in P45-53 rats. The effect of stress at an early age on memory and the HPA axis, as well as brain structures involved in memory processes in adulthood are well studied (Krugers and Joëls, 2014; Schroeder et al., 2018; Bonapersona et al., 2019; Cordier et al., 2021), but information on the effects of neonatal pain stress on memory and the participation of the HPA axis in this process is very meager. Our work is the first, as far as we know, aimed at studying the effects of early-life inflammatory pain on spatial learning and memory, and the HPA reactivity at different age intervals within the adolescent period. It was also important in our study to include male and female rats, as very few studies have included rats of both sexes in adolescence, and our results show clear differences in the effects in males and females that might be accounted for by different developmental trajectories during adolescence. The limitation of the work was that we analyzed corticosterone not after MWM, but after the further stress of the forced swim, to determine the reactivity of the HPA axis in Formalin and Control rats. We also conducted that assay only once, and thus did not evaluate the dynamics of corticosterone change. It will be interesting to investigate changes in these behavioral and endocrine systems in adult rats exposed to inflammatory neonatal pain to determine if the age and sex differences that we identified here continue into adulthood or are unique features of the adolescent period.

## Data Availability Statement

The raw data supporting the conclusions of this article will be made available by the authors, without undue reservation.

## Ethics Statement

All procedures were approved by the Local Ethics Committee for Animal Experiments of the I. P. Pavlov Institute of Physiology, Russian Academy of Sciences (Saint Petersburg, Russia) and followed the guidelines published by the Committee for Research and Ethical Issues of the IASP on ethical standards for investigations of experimental pain in animals.

## Author Contributions

IB and VM: experimental design. IB, VM, and EV: collection of data and conduction of statistical analyses. IB, VM, EV, and GB: interpretation and analysis of data, participation in the drafting and revising of the manuscript, and reviewing and approving the final submitted manuscript.

## Conflict of Interest

The authors declare that the research was conducted in the absence of any commercial or financial relationships that could be construed as a potential conflict of interest.

## Abbreviations

The HPA axis: the hypothalamo-pituitary-adrenocortical system
the HPG axis: the hypothalamus-pituitary-gonadal system
MWM: the Morris water maze
CFA: complete Freund’s adjuvant
GD: gestational day
P1: postnatal day1
P2: postnatal day 2
PVN: paraventricular nucleus
CA1: hippocampus
GR: glucocorticoid receptor
CRH: corticotrophin-releasing hormone
ACTH: adrenocorticotropic hormone
NMDA receptor: the N-methyl -D-aspartate receptor
PFC: prefrontal cortex.
