# Supplementary material for: The Long-Term Effects of Neonatal Inflammatory Pain on Cognitive Function and Stress Hormones Depend on the Heterogeneity of the Adolescent Period of Development in Male and Female Rats
Source: Front Behav Neurosci. 2021 Jul 21;15:691578. doi: 10.3389/fnbeh.2021.691578 (PMC8334561; doi:10.3389/fnbeh.2021.691578)
Supplement: Supplementary file 1 [file Table_1.DOCX]

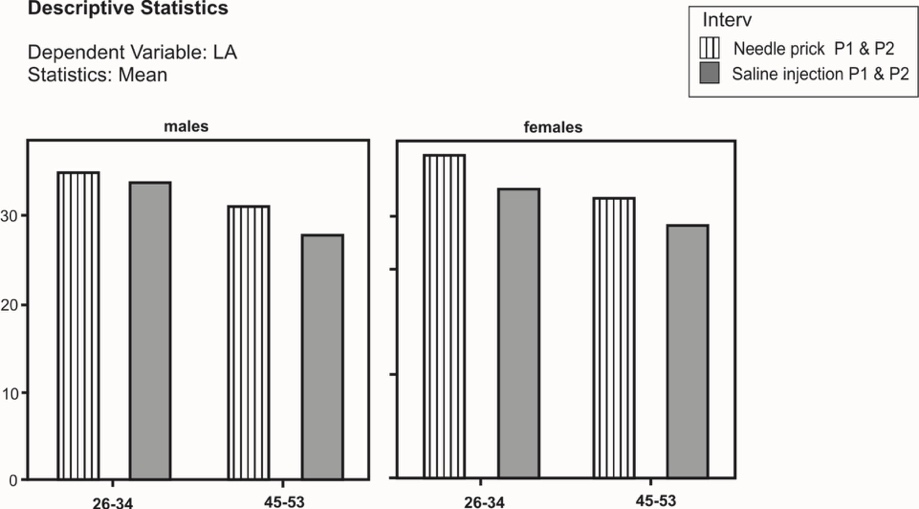


**SPATIAL LEARNING**

**Supplemental Figure 1.** Mean latency (in seconds) to find the platform in the first four training trials for the first training day of spatial learning in needle prick and saline injection male and female rats of early (P26-34) and late (P45-53) age groups

For these learning data, tests of between-subjects effects: age F(1,32) = 6,4, p = 0,017, η2 = 0,166, sex F(1,32) = 0,12, p = 0,732, η2 =0,004 and exposure F(1,32) = 1,7, p = 0,203, η2 =0,105. Interactions between factors and posthoc analysis found no differences in the latency to find the platform between the single needle prick (males n=5, females n=5) and saline injection (males n=5, females n=5).

**SPATIAL MEMORY**

**The main effects f**or memory data, mixed ANOVA, factors memory (stm 26-34 – stm 45-53, ltm 26-34 – ltm 45-53), age, sex, exposure showed no differences between single needle prick and saline injection: age F(2,31) = 50, p < 0,001, η2 =0,763, sex F(2,31) = 13,8, p < 0,001, η2 = 0,470, exposure F(2,31) = 0,073, p = 0,930, η2 =0,005, age * sex F(2,31)=7,866, p=,002, η2=,337, age * exposure F(2,31)=,098, p=,907, η2=,006, sex * exposure F(2,31)=,170, p=,844, η2=,011, age * sex * exposure F(2,31)=,104, p=,901=η2=,007.

stm - short-term memory

ltm - long-term memory


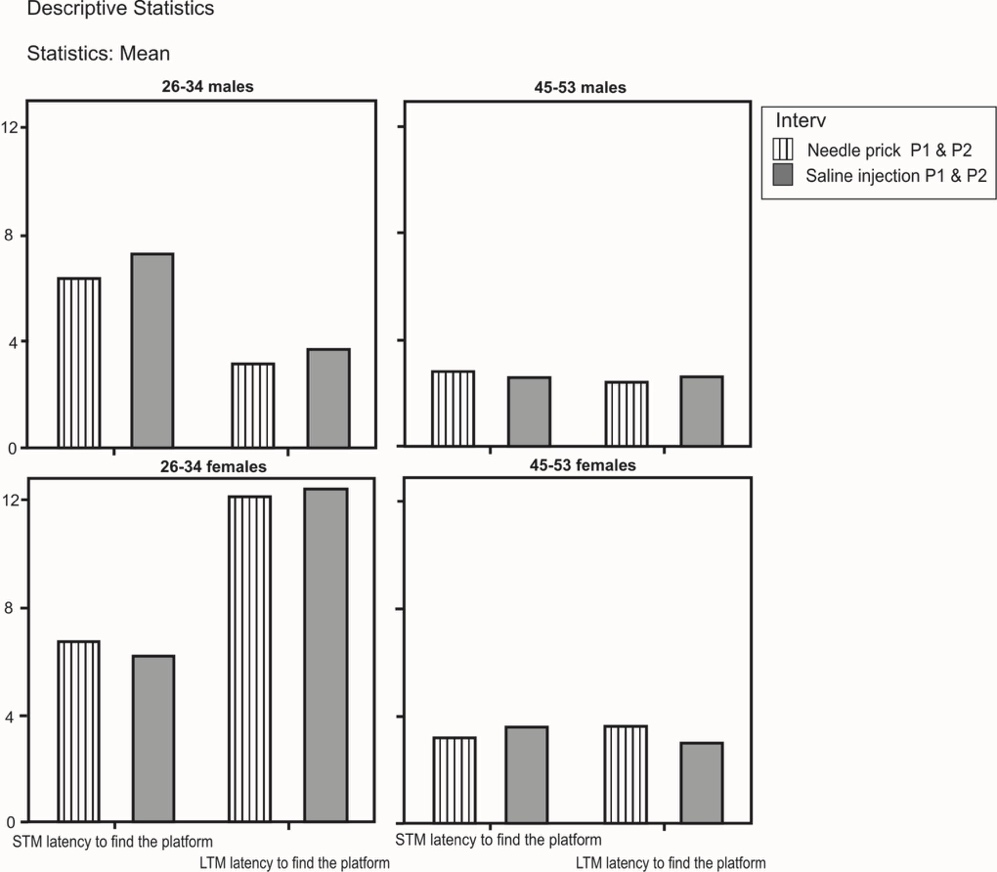


**Supplemental Figure 2.** Mean latency (in seconds) to find the platform for short-term (STM) and long-term (LTM) spatial memory in needle prick and saline injection male and female rats of early (P26-34) and late (P45-53) age groups.


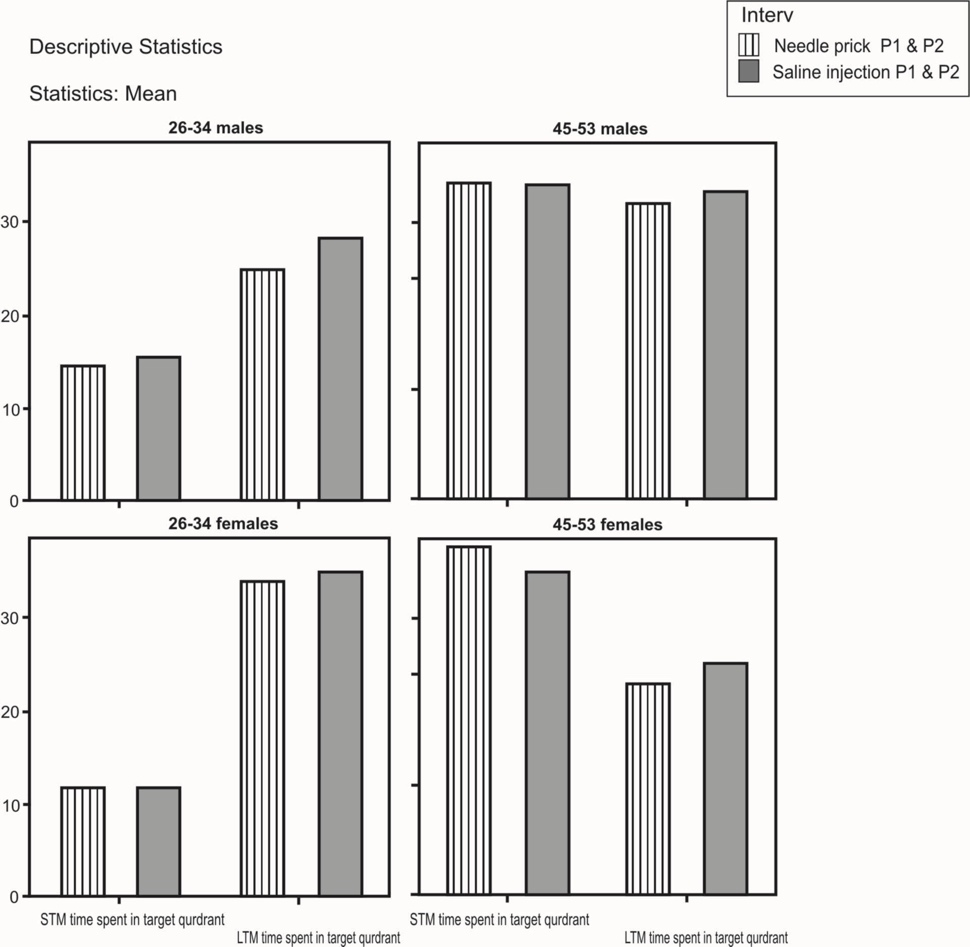


**Supplemental Figure 3.** Mean time (in seconds) in target quadrant for short-term (STM) and long-term (LTM) spatial memory in needle prick and saline injection male and female rats of early (P26-34) and late (P45-53) age groups.

**The main effects are separate for latency to find the platform and duration of being in the target quadrant**

|  | **Latency to find the platform** | **Time spent in target quadrant** |
| --- | --- | --- |
| **Univariate Tests** | **Tests of Within-Subjects Effects** |  |
| **mem** | F(1,32)= 1,01, p=0,321, η^2^ = 0,031 | F(1,32)= 16,77, p<0,001, η^2^ = 0,344 |
| **mem * Age** | F(1,32)= 1,66, p=,207, η^2^ = 0,049 | F(1,32)= 86,06, p<0,001, η^2^ = 0,729 |
| **mem * Sex** | F(1,32)= 18,64, p<0,001, η^2^ = 0,368 | F(1,32)= 0,002, p=0,966, η^2^ = 0,000 |
| **mem * Interv** | F(1,32)= 0,002, p=0,963, η^2^ = 0,000 | F(1,32)= 1,02 p=0,320, η^2^ = 0,031 |
| **mem * Age * Sex** | F(1,32)= 17,841, p<0,001, η^2^ = 0,358 | F(1,32)= 18,40 p<0,001, η^2^ = 0,365 |
| **mem * Age * Interv** | F(1,32)= 0,054, p=0,817, η^2^ = 0,002 | F(1,32)= 0,08, p=0,784, η^2^ = 0,002 |
| **mem * Sex * Interv** | F(1,32)= 0,001, p=0,970, η^2^ = 0,000 | F(1,32)= 0,04, p=0,838, η^2^ = 0,001 |
| **mem * Age * Sex * Interv** | F(1,32)= ,38, p=0,543, η^2^ = 0,012 | F(1,32)= 0,37, p=0,545, η^2^ = 0,012 |
|  | **Tests of Between-Subjects Effects** |  |
| **Age** | F(1,32)= 80,67, p<0,001, η^2^ = 0,716 | F(1,32)= 25,107, p<0,001, η^2^ = 0,440 |
| **Sex** | F(1,32)= 28,39, p<0,001, η^2^ = 0,470 | F(1,32)= ,041, p=0,840, η^2^ = 0,001 |
| **Interv** | F(1,32)= 0,06, p=0,802, η^2^ = ,002 | F(1,32)= ,082, p=0,777, η^2^ = 0,003 |
| **Age * Sex** | F(1,32)= 14,1, p=0,001, η^2^ = 0,305 | F(1,32)= 1,87, p=0,181, η^2^ = 0,055 |
| **Age * Interv** | F(1,32)= 0,13, p=0,723, η^2^ = 0,004 | F(1,32)= 0,068, p=0,796, η^2^ = 0,002 |
| **Sex * Interv** | F(1,32)= 0,29, p=0,595, η^2^ = 0,009 | F(1,32)= 0,055, p=0,816, η^2^ = 0,002 |
| **Age * Sex * Interv** | F(1,32)= 0,19, p=0,669, η^2^ = 0,006 | F(1,32)= 0,025, p=0,876, η^2^ = 0,001 |

Posthoc analysis found no differences in the latency to find the platform and time spent in target quadrant between the single needle prick (males n=5, females n=5) and saline injection (males n=5, females n=5).


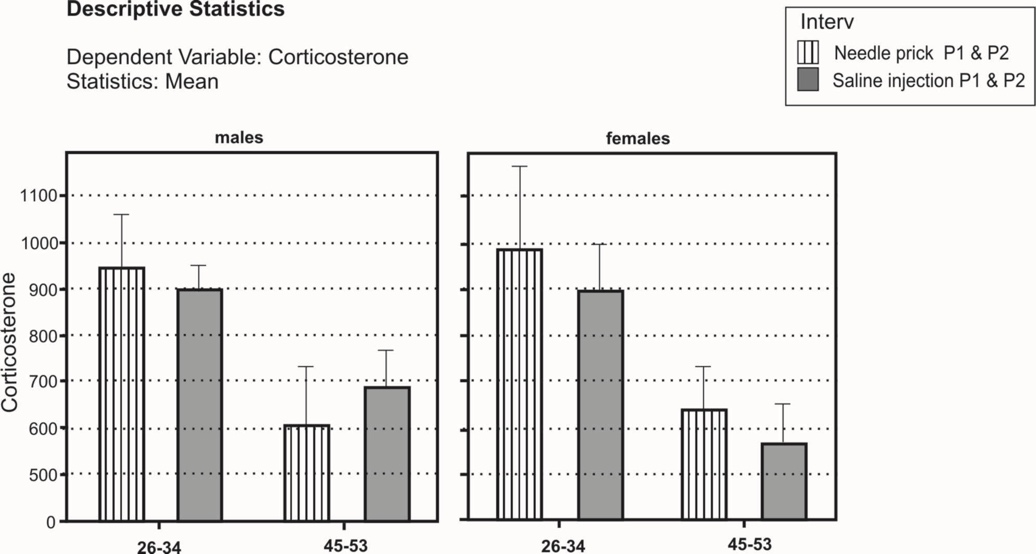


**Supplemental Figure 4.** Mean corticosterone (nmol/L) levels in blood plasma in response to forced swimming in needle prick and saline injection male and female rats of early (P26-34) and late (P45-53) age groups

Three-factor dispersion analyses ANOVA showed no differences between needle prick and saline injection in corticosterone of adolescent rats. Factors: group (early/late), sex, exposure (needle prick/saline). Main effects and interactions are not significant: sex F(1,32) =0,001, p = 0,979, exposure F(1,32) =0,029, p = 0,865, group * sex F(1,32) = 0,016, p =0,901, group * exposure F(1,32) = 0,383, p = 0,540, sex * exposure F(1,32) = 0,198, p = 0,659, group * sex * exposure F(1,32) = 0,025, p= 0,876.
